# Effect of parecoxib in the treatment of postoperative cognitive dysfunction: A systematic review and meta-analysis

**DOI:** 10.1097/MD.0000000000013812

**Published:** 2019-01-04

**Authors:** Song Huang, Haijun Hu, Yue-Hong Cai, Fuzhou Hua

**Affiliations:** a Anesthesia Department, The Second Affiliated Hospital of Nanchang University, Min De Road; b Ophthalmology Department, Jiangxi Provincial People's Hospital, Nanchang, PR China.

**Keywords:** cognitive dysfunction, inflammatory cytokine, parecoxib, randomized controlled trial

## Abstract

**Background:**

Parecoxib is a selective cyclooxygenase (COX)-2 inhibitor widely used as an analgesia technique in perioperative period for its potent anti-inflammatory and analgesic effects. However, litter is known about its effect on postoperative cognitive dysfunction (POCD). The purpose of this meta-analysis of randomized controlled trials (RCTs) was to evaluate the effect of parecoxib in the treatment of postoperative cognitive dysfunction.

**Methods:**

We searched PubMed, Cochrane Library and Embase databases for relevant studies up to October 2017. We selected fixed-effect model for analysis of data heterogeneity. Statistical analyses were performed by using Review Manager Version 5.3 for Windows.

**Results:**

Four RCTs with 904 patients that underwent surgical operations were included. The meta-analysis demonstrated parecoxib could significantly decrease the incidence of POCD on postoperative day 1, day 3, day 5, and day 7 when compared with control treatment; IL-6 and S100β concentrations were lower up to postoperative day 2. The consumption of morphine, fentanyl and tramadol in parecoxib groups were lower than control groups.

**Conclusion:**

Our meta-analysis suggested that the administration of Parecoxib was effective in treating early POCD within 7 days and reducing IL-6 and S100β concentrations within 2 days after operations. Nevertheless, our current study with some limitations such as the small sample size only provided limited quality of evidence, confirmation from further meta-analysis with large-scale, well-designed RCTs is required.

## 1 Introduction

Postoperative cognitive dysfunction (POCD) is a common situation that may occur after any sort of surgery and defined by a drop in cognitive domain on a set of neuropsychological tests before and after anesthesia and surgery.^[1]^ POCD may have many clinical implications, such as poorer neurocognitive function, worse quality of life, greater risk of medication nonadherence and poorer clinical outcomes.^[2,3]^ Age, severity of surgery, duration of anesthesia, the stress response, inflammatory and postoperative pain are the risk of POCD, but the pathophysiology and etiology of POCD are relatively unknown.^[4–6]^

Postoperative pain increases the risk of POCD.^[7]^ There are many evidence suggests that inflammatory process caused by surgical trauma is a pivotal role in the initiation of POCD.^[8,9]^ Preclinical studies also indicated that the postoperative memory deficit is paralleled by increasing levels of cytokines in the plasma and hippocampus, and interrupting the inflammatory process may mitigate memory dysfunction.^[10,11]^ Under normal quiescent conditions, the immune system is activated by environmental and psychological stimuli and secretes low levels of proinflammatory cytokines, such as interleukin-1 (IL-1), IL-6, and tumor necrosis factor (TNF)-α, and inflammatory mediator, such as prostaglandins which positively regulates the remodeling of neural circuits, promotes memory consolidation and promotes neurogenesis. However, under strongly activated conditions, such as infection or surgical trauma, glia and other brain immune cells change their morphology and secrete high levels of proinflammatory cytokines and prostaglandins. The overproduction of these inflammatory mediators creates a neurotoxic response, produces direct detrimental effects on memory, neural plasticity and neurogenesis, eventually leads to POCD.^[12,13]^ The expression of serum S100β and neuron-specific enolase (NSE) were reported to correlate with POCD.^[14,15]^

Parecoxib is a selective cyclooxygenase (COX)-2 inhibitor drug and increasingly used as analgesic in postoperative analgesia.^[16,17]^ Furthermore, parecoxib could attenuate inflammation by decreasing the formation of prostaglandin by inhibiting of COX-2 activity in both peripheral and central tissues. Perioperative administration of parecoxib after total knee arthroplasty attenuates patients’ pain by decreasing local inflammation factors.^[18]^ Surgery is associated with central neuroinflammation response and pain stress in humans. Animal studies suggested that parecoxib may be a promising candidate for the treatment of neuroinflammation and cognitive decline caused by surgical trauma.^[19,20]^ However, it remains unclear that administration of parecoxib in the perioperative period can decrease the incidence of POCD in clinical trials. Therefore, the aim of our current systematic review and meta-analysis is to evaluate the clinical effect of parecoxib on POCD based on randomized controlled trials (RCTs).

## 2 Material and methods

This meta-analysis was performed in accordance with the Preferred Reporting Items for Systematic Review and Meta-Analyses (PRISMA) guidelines.^[21]^ Ethical approval for this study was unnecessary because it was a review of existing literature and didn’t involve any handling of individual patient data.

### 2.1 Search strategy

Pubmed, Cochrane Library and Embase electronic databases were searched to identify RCTs compairing parecoxib with control for attenuating postoperative cognition in patients who underwent surgery. Two authors independently searched for relevant studies through October 2017. The search strategies for PubMed: Search ((((((((((((((((Inhibitors, Cyclooxygenase 2) OR Cyclooxygenase-2 Inhibitors) OR Inhibitors, Cyclooxygenase-2) OR Coxibs) OR COX-2 Inhibitors) OR COX 2 Inhibitors) OR Inhibitors, COX-2) OR COX2 Inhibitors) OR Inhibitors, COX2)) OR “Cyclooxygenase 2 Inhibitors”[Mesh])) OR (((((((N-(((5-methyl-3-phenylisoxazol-4-yl)-phenyl)sulfonyl)propanamide) OR N-(((Me-P)-P)S)P) OR parecoxib sodium) OR N-(((5-methyl-3-phenylisoxazol-4-yl)-phenyl)sulfonyl)propanamine, sodium salt) OR Dynastat)) OR “parecoxib” [Supplementary Concept]))) AND (((((((((Disorder, Cognition) OR Disorders, Cognition) OR Overinclusion)) OR (((((Cognitions) OR Cognitive Function) OR Cognitive Functions) OR Function, Cognitive) OR Functions, Cognitive)) OR “Cognition”[Mesh]) OR “Cognition Disorders”[Mesh])) AND ((randomized controlled trial[Publication Type] OR randomized OR placebo))). The bibliographies of related systematic reviews and clinical guidelines were also searched. In addition, the reference section for each study was also searched.

### 2.2 Inclusion and exclusion criteria

We made our inclusion and exclusion criteria in adherence to the PICOS principle. It was published in English. *P*: subjects enrolled in our systematic review were patients undergoing surgical operations and no restriction on race, age, and gender was imposed; *I*: patients in parecoxib groups were treated with parecoxib intravenously before or/and after surgeries; *C*: control groups received a placebo administration of normal saline; the volume of normal saline must be the same as parecoxib administration; *O*: the primary outcome measures included incidence of POCD; secondary outcomes were IL-6, IL-1β, TNF-α, S100β, and NSE; S: study design was restricted to RCTs. Case reports, case series, book chapters, and editorials were excluded, and only the latest research or the research with complete information was included.

### 2.3 Data extraction

Two authors screened each article independently and were blinded to the finding of the other reviewer. Following the prespecified inclusion criteria, 2 authors performed a rigorous screening to identify eligible articles. Data were collected from these selected articles using a standardized data collection sheet, which included first author, country, year of the publication, study design, cohort sizes, demographic characteristics of participants in different groups, details of intervention and control, and main outcomes. (Comment 2) The kappa value for the selection and data extraction is 0.713. Discrepancies between 2 authors were resolved through discussion until reaching a general consensus. The third author was sought for opinions if a consensus could not be reached.

### 2.4 Risk of bias assessment

We used the Cochrane Collaboration's tool to assess the risk of bias among our included studies. The Cochrane Collaboration's tool was based on 7 items: random sequence generation, allocation concealment, blind of participants and personnel, blinding of outcome assessment, incomplete outcome data, selective reporting, and other sources of bias.^[21]^ (Comment 6) One of 4 studies^[22]^ was registered on Chinese Clinical Trial Registry, the other 3 studies were only approved by the Ethics Committee of their Hospital and not registered. Two authors judged the risk of bias among studies independently; the results were compared afterward. In case of disagreements about the risk of bias judgment, discussion was conducted until a consensus was reached.

### 2.5 Data synthesis

About the incidence of POCD, risk ratio (RR) and the associated 95% confidence interval (95% CI) were calculated using the Rev Man 5. 3 (Copenhagen: the Nordic Cochrane Centre, the Cochrane Collaboration, 2014). Mean difference (MD) and the associated 95% CI were calculated for continuous variables using the same methodology. Before the combination of data from individual study, the chi-squared test and the Higgins *I*^2^ test were used to assess the heterogeneity among studies (*P* > .1 and *I*^2^ indicate acceptable heterogeneity). A fixed-effect model was used for statistical analysis when there was no significant statistical heterogeneity (*P* > .1 and *I*^2^<50%). Otherwise, a random- effect model statistical method was employed. (15,16) Subgroup analysis was performed and publication bias was evaluated using funnel plots.

## 3 Results

### 3.1 Characteristics of identified studies

An initial literature search yielded a total of 108 potentially relevant citations including 21 from PubMed, 75 from EMBASE and 12 from Cochrane Library; 35 duplicated were deleted. At the stage of titles and abstracts screen, 56 articles were excluded because they were not related with parecoxib in the treatment of POCD, and the remaining 17 articles were retrieved for a full-text review. Finally, 4 studies met our predetermined inclusion criteria.^[22–25]^ All included studies were the randomized controlled trials. All the included studies were conducted in China and published from 2013 to 2017; These 4 RCTs included a total of 904 elderly patients that underwent surgical operations: 452 in the parecoxib groups and 452 in the control groups. All of the prospective RCTs enrolled patients that underwent orthopedic operation. The characteristics of included studies were summarized in Table 1.

**Table 1:**
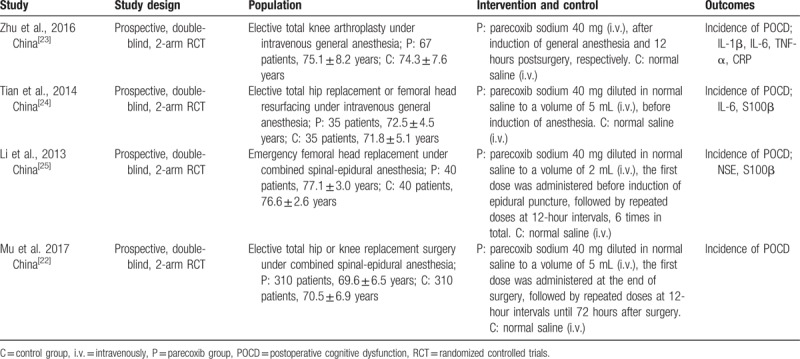
Characteristics of included studies.

### 3.2 Definition of POCD

Zhu and colleagues^[23]^ assessed the neuropsychological state of enrolled patients using a brief battery of neuropsychological test, any patient demonstrated deficit in 2 or more tests was considered as having POCD. The other three studies^[22–24]^ determined the incidence of POCD using MMSE scale, POCD was defined if the difference between post- and preoperative MMSE score was equal or larger than the standard deviation of preoperative MMSE scores

**Figure 1:**
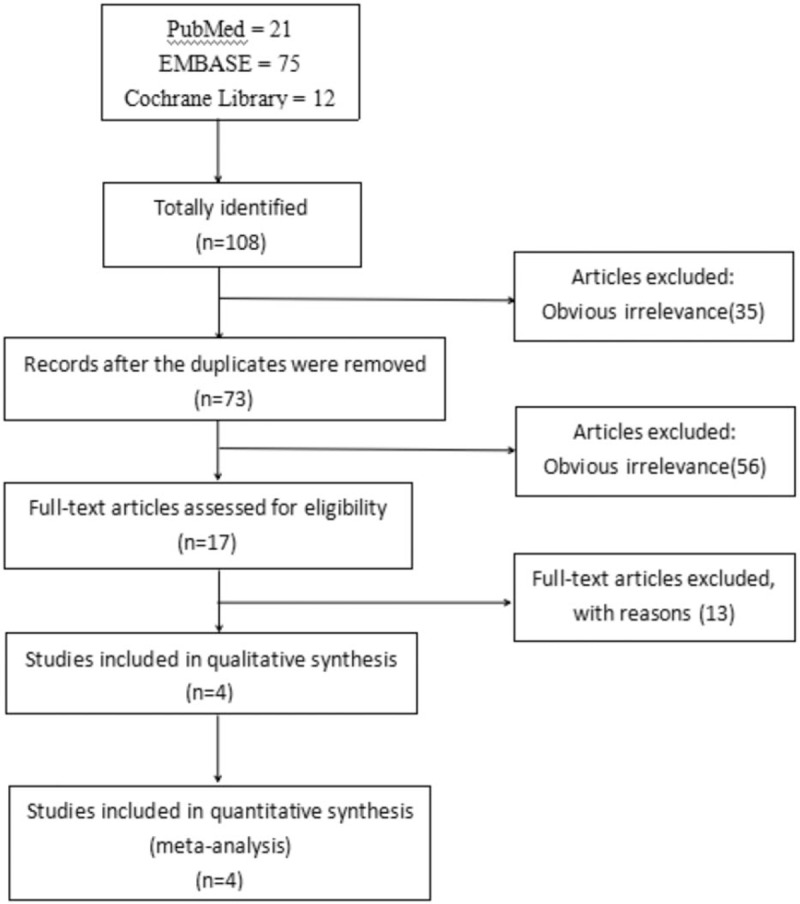
Flowchart of literature search and study selection.

### 3.3 Risk of bias assessment

**Figure 2:**
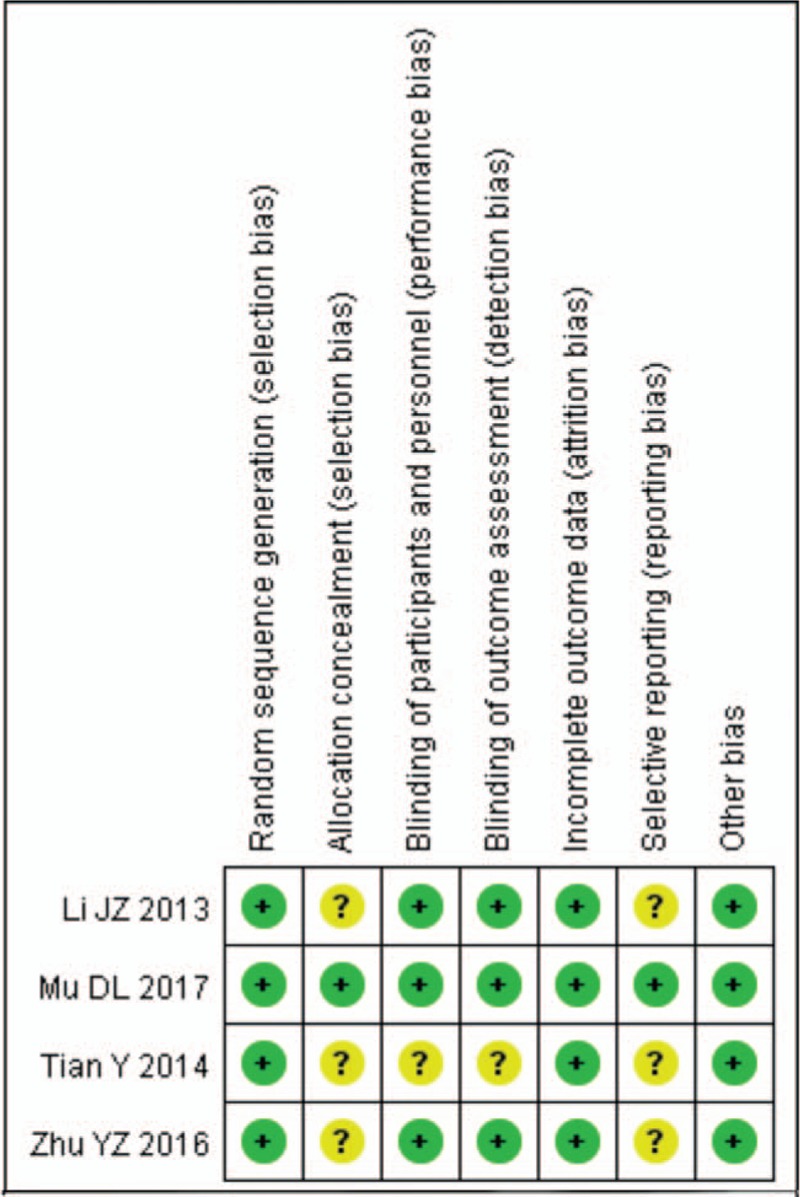
Risk of bias summary: review authors’ judgements about each risk of bias item for each included study.

**Figure 3:**
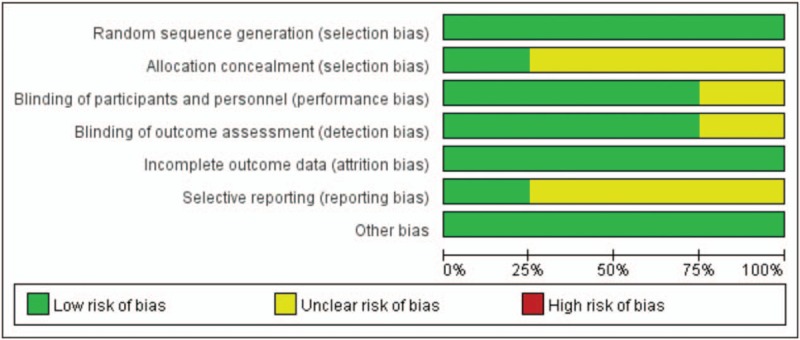
Risk of bias graph: review authors’ judgements about each risk of bias item presented as percentages across all included studies.

All the studies included the suggested randomization and reported the method of random sequence generation. Three studies reported the procedure of allocation concealment and double-blinding: All the study drugs were prepared according to the randomization code and were identical in appearance and provided in syringes of the same size and brand. Two studies reported number of drop-outs. When it comes to selective reporting bias, all studies were judged low risk of bias because we only included studies which reported incidence of POCD. The judgment of risk of bias was presented in Figures 2 and 3.

### 3.4 Incidence of POCD

**Figure 4:**
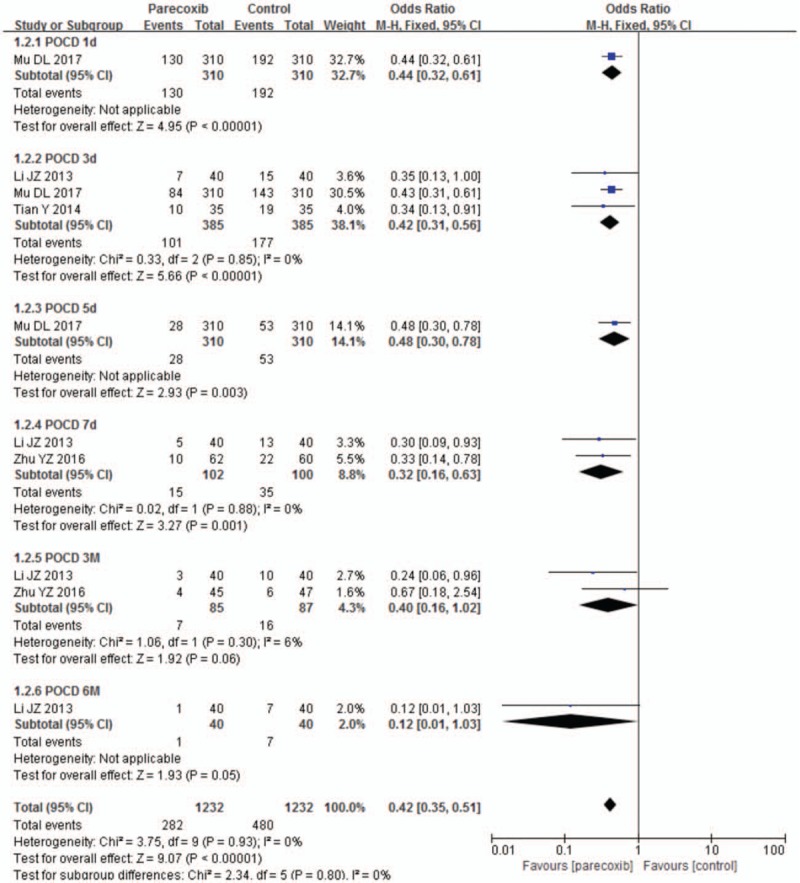
Forest plot of parecoxib versus control: incidence of POCD. POCD = postoperative cognitive dysfunction.

All the included studies measured incidence of POCD as outcome assessment. As there was no obvious heterogeneity, fixed-effect model was utilized for statistical analysis. Subgroup analysis was conducted according to the different timing of neuropsychological tests. The meta-analysis showed that parecoxib could significantly decrease the incidence of POCD on postoperative day 1 (RR 0.44, 95% CI 0.32, 0.61; *P* < .00001), day 3 (RR 0.42, 95% CI 0.31, 0.56; *I*^2^ = 0; *P* < .00001), day 5 (RR 0.48, 95% CI 0.30, 0.78; *P* = .003) and day 7 (RR 0.32, 95% CI 0.16, 0.63; *I*^2^ = 0, *P* = .001) when compared with control treatment; but the meta-analysis showed no statistical difference of incidence of POCD between parecoxib and control groups on postoperative month 3 (RR 0.40, 95% CI 0.16, 1.02; *I*^2^ = 6%, *P* = .06) and month 6 (RR 0.12, 95% CI 0.01, 1.03; *P* = .05) (Fig. 4).

### 3.5 Secondary outcomes

In addition to the incidence of POCD, IL-6 and S100β concentrations were also measured by our included studies. Baseline similarities in parecoxib and control groups were described in all the studies. IL-6 and S100β concentrations increased significantly within the 48 postoperative hours. All these aforementioned laboratory indicators were significantly lower in parecoxib groups than control groups from the end of operation to the 48 postoperative hours. There were no significant differences of IL-6 and S100β on the 72 postoperative hours. (Table 2)

**Table 2:**
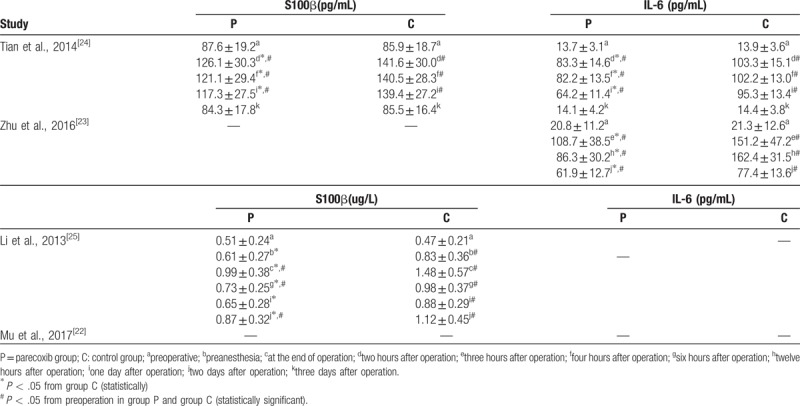
Secondary outcomes reported by included studies.

### 3.6 Other outcomes

Two studies^[22,24]^ reported clinical characteristics and postoperative situation, such as consumption of analgesic and nausea. In the study conducted by Mu et al, the incidence of nausea and vomiting, NRS pain score at rest and cumulative morphine consumption were lower in parecoxib group than control group. Tian et al reported that consumption of fentanyl and tramadol during PCIA and remedial amount of fentanyl used were significantly lower in the parecoxib group than in the control group. (Table 3)

**Table 3:**
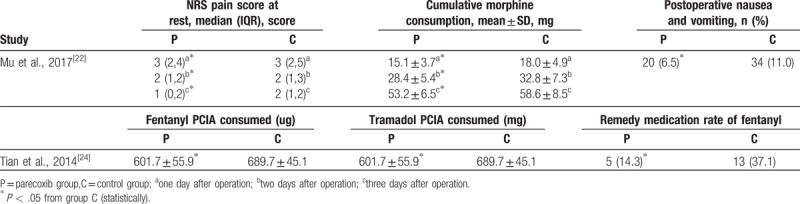
Other outcomes reported by included studies.

### 3.7 Publication bias

**Figure 5:**
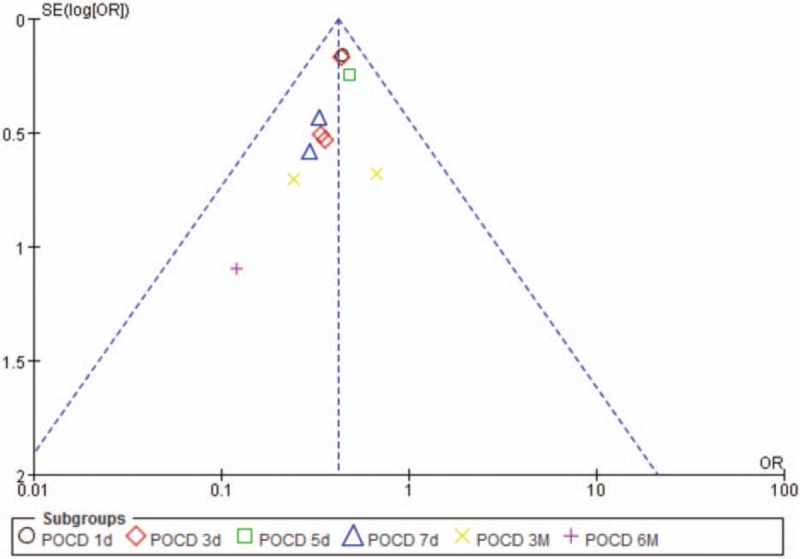
Funnel plot of parecoxib versus control: incidence of POCD. POCD = postoperative cognitive dysfunction.

Publication bias was explored via funnel plots (Fig. 5). The funnel plots presented asymmetry, indicating publication bias.

## 4 Discussion

To the best of our knowledge, this is the first systematic review and meta-analysis to assess the clinical effect of parecoxib in the treatment of patients with early POCD. Four RCTs involving a total of 904 patients were identified by our current work. Based on the finding of this study, parecoxib was effective in reducing the incidence of POCD and inhibiting the release of inflammatory cytokines. Compared with control groups, the consumption of postoperative analgesia and postoperative nausea and vomiting were lower in parecoxib groups.

Inflammation play a critical role in the pathogenesis of POCD.^[26,27]^ Peripheral pro-inflammatory cytokines could cross the blood-brain barrier (BBB) via saturable transporters^[28]^ or by passive diffusion through spaces between vascular endothelial cells,^[29]^ and could influence cognitive function by interfering with neuronal activity, affecting the function of synaptic connections, causing neuron toxicity and causing neuron degeneration, finally resulted in POCD.^[26]^ IL-6 is one of the pro-inflammatory cytokines, and it is associated with cognitive impairment.^[30,31]^ Our included studies observed a significant increase of IL-6 after operation, and the concentration of IL-6 returned to normal on the third day after operation. The incidence of POCD and concentration of IL-6 in parecoxib group were significantly lower than control group within the first 2 days after operation, this suggested that parecoxib may attenuate POCD by inhibiting the release of IL-6.

The blood-brain barrier (BBB) dysfunction may lead to the release of molecules which usually remain confined to brain tissues, one of these molecules is S100β (S100 calcium-binding protein β) that has been considered a biomarker of cognitive impairment.^[32]^ S100β belongs to the family of calcium-binding proteins. It was expressed primarily by astrocytes and found both intra- and extra-cellularly in brain tissue.^[33]^ Studies have shown that enhanced S100β upregulated the cyclooxygenase-2 expression by way of RAGE in microglia,^[34]^ and in those with Alzheimer disease cyclooxygenase-2 inhibitor impeded neuro-inflammation and induced amelioration of cognitive function.^[19]^ Our included studies observed the incidence of POCD and concentration of S100β in parecoxib group were significantly lower than control group within the first 2 days after operation, this suggested that parecoxib may attenuate POCD by inhibiting the generation of S100β which might play a critical role in driving the pathogenesis of POCD.

Postoperative pain is intensified by sensitization of the pain receptors in the spinal cord and brain^[2]^ and considered to be a risk factor for POCD as the areas of the brain involved in pain perception and cognitive overlap.^[35]^ Morphine,^[36]^ tramadol and fentanyl^[37]^ were also the risk factors of POCD. Parecoxib is usually used as nonopioid analgesia in perioperative period. Our included studies demonstrated parecoxib could attenuate POCD by relieving perioperative pain and reducing the consumption of morphine, tramadol, and fentanyl.

There were several limitations in our systematic review. Firstly, only 4 RCTs involving 904 patients were selected by us; the sample-sizes of these studies were relatively small. Moreover, we searched only three electronic databases and didn’t search for unpublished trials. Finally, all these trials were conducted in China, these works had only been performed in a Chinese population, clinical studies within western culture to evaluate the effect of parecoxib are encouraged.

## 5 Conclusion

In summary, parecoxib showed remarkable effect in reducing the incidence of POCD and could significantly reduce the concentrations of IL-6 and S100β from the end of operation to postoperative day 2. Further international studies are need to conduct and the mechanisms involved in the mitigation of POCD by parecoxib are need to understand.

## Author contributions

**Conceptualization:** Fuzhou Hua, Haijun Hu.

**Data Curation:** Song Huang, Haijun Hu.

**Formal analysis:** Song Huang.

**Investigation:** Haijun Hu.

**Methodology:** Haijun Hu.

**Project administration:** Fuzhou Hua.

**Software:** Song Huang.

**Supervision:** Yuehong Cai.

**Validation:** Yuehong Cai.

**Visualization:** Yuehong Cai.

**Writing – original draft:** Song Huang.

**Writing – review & editing:** Fuzhou Hua.
